# Can hemophilia be cured? It depends on the definition

**DOI:** 10.1016/j.rpth.2024.102559

**Published:** 2024-08-27

**Authors:** Lieke Baas, Rieke van der Graaf, Karina Meijer

**Affiliations:** 1 Department of Bioethics and Health Humanities, Julius Center for Health Sciences and Primary Care, University Medical Center Utrecht, Utrecht University, Utrecht, the Netherlands; 2 Department of Hematology, University Medical Center Groningen, University of Groningen, Groningen, the Netherlands

**Keywords:** ethics, genetic therapy, hemophilia A, hemophilia B

## Abstract

Over the years, the palette of treatment options for hemophilia has grown extensively, leading to an increased life expectancy and quality of life for people living with hemophilia. Nonetheless, it is frequently emphasized that none of the current treatment modalities provides a “cure.” It is therefore hoped that innovative treatments such as gene therapy may bridge this void. However, the precise definition of a “cure” for hemophilia remains unclear. In this review, we show how the concept of cure is currently used in the field of hemophilia. We then relate the discussion on cure to debates surrounding the classification of hemophilia and philosophical debates on the concepts of health and disease.

## Essentials

• Many expect that gene therapy will cure hemophilia, but it is unclear what is meant by “cure.”
• We review the use of “cure” in hemophilia and relate this to the concepts of health and disease.
• “Cure” is used ambiguously and can refer to both factor expression and quality of life.
• The field should evaluate the need for a single definition and who has a role in articulating it.

## 1 Introduction

Hemophilia is a rare, congenital bleeding disorder characterized by a lack of clotting factor (F)VIII (hemophilia A) or FIX (hemophilia B). If left untreated, the disorder leads to spontaneous or trauma-induced bleeding into muscles and joints [1]. Hemophilia can also lead to unstoppable bleeding in essential organs after trauma or surgery, which means that without treatment, many people living with hemophilia are likely to die during childhood or early adulthood [2]. Over the past 6 decades, hemophilia has transitioned from a fatal disease whereby patients were not expected to live longer than 20 to 30 years to a manageable disorder with a life expectancy close to that of the general male population [3].

Nowadays, people living with hemophilia in resource-rich settings have access to a variety of treatment options. Standard of care consists of prophylaxis with clotting factor replacement products, requiring intravenous injections several times a week [4]. From the 2010s, clotting factor products with an extended half-life have reached the market, thereby decreasing the number of injections required and thus decreasing the burden of treatment [3]. More recently, the nonreplacement product emicizumab has become available for people with hemophilia A, which can be injected subcutaneously and requires less frequent injections, once every 1 to 4 weeks. Other nonreplacement or rebalancing products for hemophilia A and B are currently in development [3].

Although this array of treatment options has increased the quality of life for people living with hemophilia, it is often stated that none of these options provides a definitive cure. Therefore, many hope that innovative treatments such as gene therapy will fill this gap [3]. At the same time, a consensus definition of a cure for hemophilia is lacking [5].

In this review, we show how the concept of cure is currently used in the field of hemophilia and relate it to the debate surrounding the classification of hemophilia that has recently unfolded. The paper consists of 3 parts. In section 2, we review the current use of the concept of “cure” for hemophilia. In section 3, we discuss the current debate surrounding the classification of hemophilia and its relevance for discussions on cure. In section 4, we relate both debates to philosophical notions of health and disease. We end by highlighting some points that require consideration in the further debate on these concepts.

## 2 A “Cure” for Hemophilia

Numerous scholars highlight that gene therapy holds the potential to cure hemophilia. At the same time, they oftentimes do not explain or clarify what they mean by “cure” (eg, [[6], [7], [8], [9]]). Some argue that “the possibility of curing, rather than simply controlling, the disease has been the holy grail of hemophilia treatment and is finally becoming a reality with gene therapy” [10]. Others mention that “a gigantic unmet need was the lack of a cure” [3]. The promise of cure appears to arise mainly in relation to gene therapy, although some authors also mention that cell therapy [11,12] or liver transplantation [13,14] may provide a cure.

### 2.1 The use of cure in the context of outcome sets

The most concrete and elaborate definitions of “cure” for hemophilia are provided as part of sets of core outcome measures for the treatment of hemophilia. Several core outcome sets for hemophilia have been developed, each with a slightly different focus [[15], [16], [17], [18], [19]]. Three of these sets incorporate cure as an outcome; these sets will be discussed below.

The coreHEM outcome set aims to determine a core set of outcomes to evaluate the efficacy, safety, comparative effectiveness, and value of gene therapy trials [18]. CoreHEM has identified the following 6 core outcomes: frequency of bleeds, factor activity level, duration of expression, chronic pain, utilization of the healthcare system, and mental health [18]. Integration of all these elements collectively constitutes a cure for hemophilia. According to the authors, this interpretation of cure encompasses the entire spectrum of the functional and social impact of living with hemophilia [18].

Another core outcome set was developed by van Balen et al. [17], focusing on health outcomes relevant to people with hemophilia. This project has identified a list of 10 health outcomes. Cure is one of these outcomes, next to the impact of the disease on life expectancy, ability to engage in normal daily activities, severe bleeding episodes, number of days lost (work or school), chronic pain, complications, sustainability of physical function, social functioning, and mental health [17]. The authors provide a specific definition of cure as follows: “complete correction of previous bleeding tendency with normalized clotting factor levels 5 years after curative treatment, requiring no further treatment (with coagulation factor or other treatments), not even for surgery or bleeding. Cure is phenotypically intended and does not include eliminating transmission of hemophilia to children or fully reverting established damage” [17].

A third outcome set, developed by Skinner et al. [19], defines a functional cure based on 7 levels, with the goal of ultimately achieving health equity for all people with hemophilia worldwide. The 7 levels are considered milestones that people living with hemophilia can achieve successively, thereby describing a progressive definition of cure. The levels are survival, minimal joint impairment, freedom from spontaneous bleeds, attaining “normal” mobility, ability to sustain minor trauma, undergo surgery or major trauma without additional intervention, and normal hemostasis [19]. In parallel to the set of outcomes defining cure, a set of patient-reported outcomes to achieve health equity was defined, matching the clinical outcomes. These patient-reported outcomes are preventing premature death, improved quality of life/participation in activities of daily living, ability to engage in low-risk activities, participation in work, career, and family without restriction, more unrestricted lifestyle, not dependent on specialized health care, and optimized health and well-being [19].

Thus, based on these outcome sets, cure can be regarded in several ways, whereby some interpretations are broader than others. Cure may be seen as a correction of the bleeding tendency [17,19], or in addition to this, also incorporates prevention of joint damage [19]. The concept is also used in a more comprehensive way, whereby it includes the absence of chronic pain and the impact of hemophilia on a person’s psychological state [18].

### 2.2 Characteristics of cure

In addition to the specific definitions developed as part of core outcome sets, several authors mention characteristics of a cure. Based on these descriptions, several aspects of a cure for hemophilia can be identified.

The first characteristic is its durability. Several authors mention that a cure is “permanent” [20,21], “definitive” [22], “lasting” [23], “lifelong” [[24], [25], [26]], or “durable” [27], or leads to “stable” [28,29] or “continuous” [30,31] expression of endogenous clotting factor. Several authors state that a cure is a single or “once and done” treatment [14,27,32,33].

Based on the argument on “durability,” there is disagreement about whether gene therapy has ever resulted in a cure. Some argue that it has, based on trial follow-up data of several months up to a few years [22,26,[34], [35], [36]]. Others argue that the current lack of knowledge about the durability of gene therapy precludes a definitive conclusion on cure [37]. Still, others argue that the declining clotting factor levels seen in a number of gene therapy trials may be considered a “near-cure” [38].

Further, several authors mention that a cure leads to endogenous factor expression [[28], [29], [30], [31],^39^,^40^]. For some, a cure entails factor expression in the normal range (50%-150%) [11,41,42]. Others state that curative levels do not have to equal normal levels or speak of a cure when clotting factor levels have been raised to the mild or moderate range or when factor levels exceed 10% [26,43,44]. It is also said that a cure leads to “symptom relief” [45] or that cure entails being free of spontaneous bleeding [33,44,46] without requiring prophylaxis [44,46].

Finally, some authors emphasize the quality of life-related aspects of a cure. For instance, they argue that because the standard of care already leads to long-term survival, a cure for hemophilia revolves around enhancing the quality of life throughout an individual’s lifespan [5]. Further, it is stated that cure goes beyond stopping and preventing bleeding and is more concerned with normalizing the lives of people living with hemophilia [47].

## 3 Cure and the Classification of Hemophilia

Over the last few years, a debate has developed regarding the classification of hemophilia [[48], [49], [50]]. Given that the concept of cure refers in some way to a transition from having hemophilia to a state of health, the debate surrounding the classification of hemophilia might be informative to better understand the concept of cure. In this section, we will summarize this debate on classification and draw lessons from it.

According to the official guidelines of the International Society on Thrombosis and Haemostasis (ISTH), “plasma procoagulation levels, rather than clinical bleeding symptoms, should be used preferentially for the classiﬁcation of hemophilia” [51]. According to this classification, plasma factor levels of <1% of normal are classified as severe, 1% to 5% as moderately severe, and >5% to 40% as mild [51].

However, recently, Thachil et al. [48] have argued that this does not do justice to clinical reality, given that coagulation factor activity levels do not always correlate with bleeding phenotype. In addition, they mention that different clotting factor assays differ in accuracy, thereby risking misdiagnosis when used as the sole source for classifying hemophilia into the categories of severe, moderate, and mild [48]. Therefore, they argue for a shift in treatment target, away from a clinicians’ perspective on the disorder to a focus on quality of life. Therefore, they propose that “those with signiﬁcant symptoms are those with bleeding that negatively impacts HRQoL [health-related quality of life] issues and require an intensiﬁcation of treatment to maintain hemostasis and keep secondary morbidities at bay” [48]. In a second piece defending their original argument, the authors state that care for people living with hemophilia should be directed toward the World Health Organization’s (WHO) definition of health as a state of complete physical, mental, and social well-being [52].

This proposal has received both critique and support, which mainly seems to focus on the question of what the goal of disease classification is [49,50,52]. Critics of revising the definitions of hemophilia severity argue that the goal of disease classification is correct prognostication and to allow patients to find and be treated by the correct physician, and therefore argue for the importance of distinguishing between prognostication and adopting an outcome-based treatment approach [49]. Supporters of a revised definition emphasize the importance of the lived experience of people living with hemophilia and the sociopolitical effects of labels, eg, regarding social policies developed based on such classifications and access to and reimbursement for treatment [48,50]. These authors do not deny the importance of classification for selecting appropriate treatment and prognostication but argue that a reclassification allows for a shift from provider-centered care to care centered on people living with hemophilia [50,52].

A similar discussion has unfolded surrounding the labeling of women and girls with a gene variant for hemophilia. Historically, women and girls have been called “hemophilia carriers.” It has been argued that because hemophilia is an X-linked disorder and, therefore, mainly affects males, there is a bias toward assuming that hemophilia carriers are asymptomatic [53]. However, there is now an increasing body of evidence that women and girls with such a gene variant experience heavy periods, joint damage, pain, and impaired quality of life [[53], [54], [55], [56]]. It has been argued that this labeling system is an example of sexism and hampers diagnosis, care, and research [54,56].

Therefore, a new nomenclature for women and girls with hemophilia has been suggested by the ISTH, which suggests using the following 5 categories: women and girls with mild, moderate, and severe bleeding, symptomatic carrier, and asymptomatic carrier [53]. The 2 “carrier” classifications are intended to describe women and girls with normal coagulation factors, acknowledging that they still may have an increased bleeding tendency. It is also suggested that the term “hemophilia carrier” should be reserved for genetic counseling [53].

These debates show how classifications of disease severity serve various purposes and uses: predicting disease progression, providing access to treatment options, acknowledging impacts on quality of life, and guiding social policies, to name a few. In addition, the discussion highlights how social influences (in the discussion surrounding women and girls with hemophilia) and progress in treatment options (in the discussion surrounding the classification of hemophilia severity) have an impact on how such concepts are interpreted and used. Lastly, the debate prompts questions about which perspectives should be incorporated into these decisions and who gets to decide on the final definition. For instance, authors in this debate draw attention to the importance of incorporating the views of both adult and child hematologists [49] and argue for the importance of the ISTH Scientific and Standardization Subcommittee in taking the lead on these decisions [49,52] or explicitly highlight their own experience as a person living with hemophilia as well as professional expertise [50].

## 4 The Concepts of Health and Disease

The questions surrounding classification that are arising now in the field of hemophilia are similar to questions surrounding the definitions of health and disease in other medical fields and debates in the philosophy of medicine. The latter debate has resulted in the development of various theories of health and disease that coexist. Three prominent theories are presented below, as they may inform the discussion surrounding a cure for hemophilia.

### 4.1 The biostatistical theory of health

The “biostatistical theory of health” (BST) is one of the most prominent theories on health. Christopher Boorse, a philosopher of medicine, claims that disease should be defined based on an assessment of the biological functioning of 1 or more organs of an organism. According to the BST, the disease is “a type of internal state which is either an impairment of normal functional ability, ie, a reduction of one or more functional abilities below typical efficiency, or a limitation on functional ability caused by environmental agents.” Following from this definition, health “is the absence of disease” [57]. In this definition, normal functioning refers to a certain part or organ’s contribution to an organism’s reproduction or survival. According to Boorse, in order to understand statistically normal functioning, we should examine the functioning of a specific age group of a sex. Importantly, according to the BST, the concepts of health and disease are merely descriptive and do not refer to any values. Boorse has described his definition of disease as corresponding to a pathologist’s use of the term rather than a clinician’s.

This theory has faced several criticisms. In particular, it has been questioned whether the most important goals of an individual are reproduction and survival [58]. Furthermore, some have argued that when statistically normal functioning is taken as a reference, “common diseases” that occur in a large part of the population, such as caries, will not be considered a disease [57].

Within the domain of hemophilia, several scholars mention that a cure entails endogenous factor expression, sometimes also referencing the decrease of bleeding symptoms and no further need for prophylaxis [[28], [29], [30], [31],^33^,^39^,^41^,^44^,^46^]. These interpretations appear to align with the BST, equating health with statistically normal functioning.

### 4.2 Holistic theories of health

Holistic theories of health refer not just to survival but to quality of life as well [59]. Such concepts of disease, therefore, include evaluative judgments. One of the most important developers of such a notion of health is the philosopher Lennart Nordenfelt. He claimed: “A is healthy if, and only if, A has the ability, given standard circumstances, to reach all his or her vital goals” [59]. Here, vital goals refer to a person’s most important goals in life. The definition of disease follows from the definition of health: “A has a disease if, and only if, A has at least one organ which is involved in such a state or process as tends to reduce the health of A. The disease is identical with the state or process itself” [59]. This theory has received criticism as well; “vital goals” are considered to be too broad and at risk of medicalization when many phenomena may be considered as “ill health” [60].

Some authors mention that a cure for hemophilia refers explicitly to quality of life-related aspects [5,47] or highlights how a cure can contribute to improving the quality of life [[17], [18], [19]]. Although our analysis indicates that the term cure is used more regularly to describe improvements in the expression of coagulation factor levels than improvements in quality of life, there has traditionally been much attention to quality of life-related aspects in hemophilia care. For instance, recently, the concept of a “hemophilia-free mind” has been proposed to guide future research and care for hemophilia [[61], [62], [63]]. This concept refers to the absence of psychological burden and constant thoughts about hemophilia, thereby freeing people living with hemophilia from the impacts of hemophilia on their behavior and daily activities [61]. From a more holistic perspective on health, this concept could very well be considered as aligning with being cured of hemophilia.

### 4.3 WHO definition and positive health

Recently, general practitioner Machteld Huber has developed a theory of “positive health” [64]. Positive health was mainly developed as an alternative to the broad WHO definition of health as “a state of complete physical, mental and social well-being and not merely the absence of disease or infirmity” [65]. Because of the requirement of “complete health” in the WHO definition, many people would be regarded as unhealthy for a significant part of their lives [64]. According to the concept of positive health, health should be defined as “the ability to adapt and self-manage in the face of social, physical, and emotional challenges” [64]. Positive health is intended to be a broad perspective on health, consisting of 6 dimensions: bodily functions, mental well-being, meaningfulness, quality of life, participation, and daily functioning [66].

From the perspective of positive health, managing aspects of daily life becomes more important, and the exact factor levels achieved might become less relevant. This aligns with recent findings from an interview study we conducted, which indicated that some Dutch people living with hemophilia A prefer emicizumab over gene therapy, at least with the current results of gene therapy. They expected to have more freedom with emicizumab and feared that after gene therapy, they would be considered to have mild hemophilia and no longer be allowed to store clotting factor at home to use in case of emergency, thereby limiting their independence [67].

## 5 Open Questions and Considerations for Discussion

This review has highlighted several topics that require further consideration in discussions surrounding cures. The first aspect concerns the durability of a cure. As discussed in the section on the characteristics of cure, it is often mentioned that cure implies a single treatment with lasting effects. However, one of the more concrete definitions of cure in an outcome set states that the effects of a cure last for 5 years after treatment. Such demarcations in time are required to measure effectiveness in trials but may be in tension with the (implicit) assumptions surrounding the concept.

Second, several findings of gene therapy trials warrant attention in this regard. To begin, the effects of gene therapy, particularly for hemophilia A, appear to decrease over time [68]. If a cure is supposed to be “lifelong” or “durable,” as many authors imply, it is thus questionable if gene therapy for hemophilia A lives up to this promise, even if it may meet the criteria of a cure as set out by a definition with a time limit of 5 years. Further, many people require immunosuppressive therapy to treat liver enzyme elevations after gene therapy [69]. If this additional treatment is required, it is questionable if gene therapy can be considered a “single treatment.” Furthermore, as gene therapy does not impact the underlying gene mutation, hemophilia will still be passed on to future generations, which might impact people’s perception of being “cured.”

Third, current debates raise the question of whether there should be an overarching, agreed-upon definition of cure. It appears that currently, many authors seem to have *interpretations* of what a cure means rather than explicit *definitions*. This is not in itself problematic. In a recent paper, we argued that different cure concepts for gene therapy may coexist as long as each concept is capable of serving the goals it is supposed to serve in a given context [70]. Nonetheless, in the debate on hemophilia classification, there appears to be a desire for one clear, agreed-upon definition. Incorporating the notion of cure in core outcome sets appears to be a move toward a standardized definition of cure. If there is a desire or need to provide an explicit definition of cure, several questions arise. Who should have a say in defining the concept, and for what contexts is the concept developed? Moreover, similar to concerns arising in the discussion surrounding classification of hemophilia, would it be problematic if a definition is used in a context for which it was not developed?

## 6 Conclusion

We have shown that the term “cure” is used ambiguously in the field of hemophilia, referring to various potential outcomes. Some of these interpretations appear mainly focused on normalization of clotting factor levels and bleeding symptoms, whereas others adopt a broader perspective incorporating quality of life. Similarly, whereas many interpretations focus on the long-lasting effects of cure, some incorporate a (limited) 5-year durability in the definition of cure. Further, we have shown how the discussions surrounding the classification of hemophilia and the concepts of health and disease relate to the discussion on cure. This review thereby raises the question of whether there should be 1 or more explicit definition(s) of cure, and if so, whose perspectives and opinions should be involved in developing this definition.
